# Inverse Association between Dietary Iron Intake and Gastric Cancer: A Pooled Analysis of Case-Control Studies of the Stop Consortium

**DOI:** 10.3390/nu14122555

**Published:** 2022-06-20

**Authors:** Giulia Collatuzzo, Federica Teglia, Claudio Pelucchi, Eva Negri, Charles S. Rabkin, Linda M. Liao, Rashmi Sinha, Lizbeth López-Carrillo, Nuno Lunet, Samantha Morais, Nuria Aragonés, Victor Moreno, Jesus Vioque, Manoli Garcia de la Hera, Mary H. Ward, Reza Malekzadeh, Mohammadreza Pakseresht, Raúl Ulises Hernández-Ramírez, Malaquias López-Cervantes, Rossella Bonzi, Michela Dalmartello, Shoichiro Tsugane, Akihisa Hidaka, M. Constanza Camargo, Maria Paula Curado, Zuo-Feng Zhang, Nadia Zubair, Carlo La Vecchia, Shailja Shah, Paolo Boffetta

**Affiliations:** 1 Department of Medical and Surgical Sciences, University of Bologna, 40138 Bologna, Italy; giulia.collatuzzo@studio.unibo.it (G.C.); federica.teglia@studio.unibo.it (F.T.); eva.negri@unibo.it (E.N.); 2 Branch of Medical Statistics, Biometry, and Epidemiology “G. A. Maccacaro”, Department of Clinical Sciences and Community Health, University of Milan, 20133 Milan, Italy; claudio.pelucchi@unimi.it (C.P.); rossella.bonzi@unimi.it (R.B.); michela.dalmartello@unimi.it (M.D.); carlo.lavecchia@unimi.it (C.L.V.); 3 Division of Cancer Epidemiology and Genetics, National Cancer Institute, Rockville, MD 20892, USA; rabkinc@mail.nih.gov (C.S.R.); linda.liao@nih.gov (L.M.L.); sinhar@mail.nih.gov (R.S.); wardm@mail.nih.gov (M.H.W.); maria.camargo@nih.gov (M.C.C.); 4 Mexico National Institute of Public Health, Cuernavaca 62100, Mexico; lizbeth@insp.mx; 5 EPIUnit—Instituto de Saúde Pública, Universidade do Porto, 4050-600 Porto, Portugal; nlunet@med.up.pt (N.L.); samantha.fmorais@gmail.com (S.M.); 6 Laboratório para a Investigação Integrativa e Translacional em Saúde Populacional (ITR), 4050-600 Porto, Portugal; 7 Departamento de Ciências da Saúde Pública e Forenses e Educação Médica, Faculdade de Medicina da Universidade do Porto, 4200-319 Porto, Portugal; 8 Consortium for Biomedical Research in Epidemiology and Public Health (CIBERESP), 28029 Madrid, Spain; nuria.aragones@salud.madrid.org (N.A.); vioque@umh.es (J.V.); manoli@umh.es (M.G.d.l.H.); 9 Cancer Epidemiology Section, Public Health Division, Department of Health of Madrid, 28035 Madrid, Spain; 10 Oncology Data Analytics Program, Unit of Biomarkers and Susceptibility, Catalan Institute of Oncology (ICO), Hospital Duran I Reynals, 08907 Barcelona, Spain; v.moreno@iconcologia.net; 11 Colorectal Cancer Group, ONCOBELL Program, Bellvitge Biomedical Research Institute (IDIBELL), 08907 Barcelona, Spain; 12 Department of Clinical Sciences, Faculty of Medicine, University of Barcelona, 08907 Barcelona, Spain; 13 Instituto de Investigación Sanitaria y Biomédica de Alicante, ISABIAL-UMH, 46020 Alicante, Spain; 14 Digestive Oncology Research Center, Digestive Disease Research Institute, Tehran University of Medical Sciences, Tehran P.O. Box 1411713135, Iran; dr.reza.malekzadeh@gmail.com (R.M.); ppakseresht@yahoo.com (M.P.); 15 Department of Agricultural, Food and Nutritional Sciences, University of Alberta, Edmonton, AB T6G 2R3, Canada; 16 Nutritional Epidemiology Group, Centre for Epidemiology and Biostatistics, University of Leeds, Leeds LS2 9JT, UK; 17 Department of Biostatistics, Yale School of Public Health, New Haven, CT 06520, USA; ruhernandez@gmail.com; 18 Facultad de Medicina, National Autonomous University of Mexico (UNAM), Coyoacán 04510, Mexico; mlopezcervantes@unam.mx; 19 Epidemiology and Prevention Group, Center for Public Health Sciences, National Cancer Center, Tokyo 104-0045, Japan; stsugane@ncc.go.jp (S.T.); akihisahidaka@hotmail.com (A.H.); 20 National Institute of Health and Nutrition, National Institutes of Biomedical Innovation, Health and Nutrition, Tokyo 162-8636, Japan; 21 Centro Internacional de Pesquisa, A. C. Camargo Cancer Center, São Paulo 01508-010, Brazil; mp.curado@accamargo.org.br; 22 Department of Epidemiology, UCLA Fielding School of Public Health and Jonsson Comprehensive Cancer Center, Los Angeles, CA 90095, USA; zfzhang@ucla.edu; 23 Icahn School of Medicine at Mount Sinai, New York, NY 10003, USA; nadia.zubair@ichan.mssm.edu; 24 Department of Medicine, University of California San Diego, San Diego, CA 92093, USA; s6shah@health.ucsd.edu; 25 Stony Brook Cancer Center, Stony Brook University, Stony Brook, NY 11794, USA

**Keywords:** gastric cancer, iron, diet, cancer subtypes, cancer subsites

## Abstract

*Background*: Inconsistent findings have been reported regarding the relationship between dietary iron intake and the risk of gastric cancer (GC). *Methods*: We pooled data from 11 case-control studies from the Stomach Cancer Pooling (StoP) Project. Total dietary iron intake was derived from food frequency questionnaires combined with national nutritional tables. We derived the odds ratios (ORs) and 95% confidence intervals (CIs) for quartiles of dietary iron through multivariable unconditional logistic regression models. Secondary analyses stratified by sex, smoking status, caloric intake, anatomical subsite and histological type were performed. *Results*: Among 4658 cases and 12247 controls, dietary iron intake was inversely associated with GC (per quartile OR 0.88; 95% CI: 0.83–0.93). Results were similar between cardia (OR = 0.85, 95% CI = 0.77–0.94) and non-cardia GC (OR = 0.87, 95% CI = 0.81–0.94), and for diffuse (OR = 0.79, 95% CI = 0.69–0.89) and intestinal type (OR = 0.88, 95% CI = 0.79–0.98). Iron intake exerted an independent effect from that of smoking and salt intake. Additional adjustment by meat and fruit/vegetable intake did not alter the results. *Conclusions*: Dietary iron is inversely related to GC, with no difference by subsite or histological type. While the results should be interpreted with caution, they provide evidence against a direct effect of iron in gastric carcinogenesis.

## 1. Introduction

Gastric cancer (GC) affects more than one million people per year and remains the fourth leading cause of cancer mortality worldwide, despite long-term decreasing trends [1]. In fact, trends of GC have been interpreted as a triumph of prevention, attributable to a decreased prevalence of *Helicobacter pylori (Hp)* and improvement in diet, as well as in the preservation and storage of foods [2,3]. While infection with *Hp* remains the main cause of GC, there is consistent evidence that smoking is a risk factor for GC [4]. High dietary intake of salt and processed meat represents other important risk factors [5], while fresh fruits, vegetables and certain micronutrients are protective against its development [6]. GC can be subdivided into cardia and non-cardia cancer by anatomic demarcations, and into two main histological types: the well-differentiated or intestinal type, and the undifferentiated or diffuse type.

Iron is an essential element for human life: it participates in a wide variety of metabolic processes, including oxygen transport, deoxyribonucleic acid (DNA) synthesis, and electron transport [7]. There are two main forms of dietary iron: heme and non-heme. Heme iron is contained only in meat, poultry, seafood, fish, and other animal foods. Non-heme iron is found in plant-based foods such as grains, beans, vegetables, fruits, nuts, and seeds and in some animal products such as eggs and dairy [8]. Accumulating evidence and metanalyses suggest that iron excess is associated with tumorigenesis in multiple human cancers, including colorectal (relative risk [RR] = 1.08, 95% confidence interval [CI] 1.00–1.17 for an increase of 1 mg/day of heme iron intake), breast (RR = 1.03, 95% CI 0.97–1.09), and lung cancer (RR = 1.12; 95% CI, 0.98–1.29) [9].

There is biologic plausibility for an association between dietary iron intake and GC. The carcinogenicity of iron has been studied in animal models, which supported the hypothesis of an inverse association, since iron deficiency may enhance *Hp* activity [10]. On the other hand, there is growing concern for the potential carcinogenic effect of excess dietary iron intake, possibly related to the effect exerted by the heme component, which is notably contained in red meat [11]. In addition, free iron (non-protein bound) may act as a prooxidant, leading to reactive oxidative species, which in turn can cause oxidative DNA damage. In addition, heme iron can lead to the production of N-nitroso compounds, which are established gastric carcinogens [12,13].

Existing studies are inconclusive regarding the association between dietary iron and GC, because of the use of different designs, different measures of iron and iron types, and methods of categorization. Accordingly, the primary objective of this study was to contribute to the literature by evaluating the association between total dietary iron intake and risk of GC within the studies of the Stomach cancer Pooling (StoP) Project, an international consortium of GC studies. A secondary objective was to further investigate this relationship according to GC anatomical subsite and histological type.

## 2. Methods

The present study is based on the StoP Project Consortium (http://www.stop-project.org/, accessed 31 March 2022), which includes 34 case-control or nested-within-cohort studies, for a total of 13,121 cases and 31,420 controls from 15 countries. The StoP Project aims at examining the role of several lifestyles and genetic determinants in the etiology of GC through pooled analyses of individual-level data after central collection and validation of the original datasets. Principal investigators signed a data transfer agreement and provided a de-identified copy of the original data set of their studies. The StoP Project received ethical approval from the University of Milan Review Board (reference 19/15 of 1 April 2015), and detailed information on the overall aims and methods was given elsewhere [14].

Twenty-one studies with >25% of missing values on the exposure (iron intake) or main confounders (dietary iron intake, tobacco smoking, socioeconomic status, dietary salt and caloric intake) were excluded. Two additional studies were excluded because of outlying values of iron intake (median either >30 mg/day or <1 mg/day, when considering that the European Food Safety Authority (EFSA) recommends mean dietary intake of 16 mg per day) [15]. We also excluded subjects with extreme values of either caloric intake (<500 and >5000 kcal/day) or extreme body mass index (BMI) (<15 and >50 kg/m^2^, for a total of 57 subjects.

The final analysis is based on 11 case-control studies with information on total dietary iron intake, including one study from Italy [16], one from Iran [17], one from Portugal [18], two from Spain [19,20], three from Mexico [21,22,23], one from Japan [24], and two from the USA [25,26]. Supplementary Table S1 shows the characteristics of each study. The analysis includes histologically confirmed GC cases; matched controls were selected based on hospital, neighborhood or population registries. Eight out of 11 studies included classification of GC location (cardia vs. non-cardia, excluding undetermined sites) and six studies included classification of histological type (intestinal vs. diffuse type, excluding undefined histology). Total dietary iron intake was calculated for each study using food frequency questionnaires (FFQ) and country-specific dietary composition tables. Iron intake information was harmonized and expressed as grams per day. Quartiles of intake were calculated across the combined distribution of controls, as well as based on study-specific distributions. Data were too sparse to allow separate analyses between heme and non-heme iron.

The final regression models included terms for study center, sex, age (≤55, 56–65, 66–75, ≥76 years), sex, tobacco smoking (never smoker, former smoker, current smoker), socioeconomic status (low, intermediate, and high categories, as defined in each study based on education, income or occupation), total caloric intake (500–1506 kcal/day, 1507–1981 kcal/day, 1982–2525 kcal/day, ≥2526 kcal/day), salt consumption (low, intermediate and high).

The analysis was repeated with and without adjustment for meat intake, in a subset of studies [16,18,19,20,21,22,23,24] with available information on this food item. We also considered additional adjustment for vegetables and fruit intake (low, intermediate and high categories, based on study-specific tertiles), BMI (18.5–24.99, 25–29.99, 30–34.99, 35–50), alcohol drinking (overall consumption: never, low—≤12 g/day, intermediate 13–47 g/day, high—>47 g/day) and family history of GC in first-degree relatives. *Hp* status was not included in the analysis given the high number of missing values. The main analysis was repeated using study-specific quartiles of iron intake.

We conducted stratified analyses to investigate the effect of dietary iron intake across strata of sex, smoking status, caloric intake, *Hp* infection (among the studies with <10% of missing information for this variable), histological type, anatomical subsite, and type of controls (hospital-based vs. population-based).

We considered the interaction between iron intake and salt consumption, as well as between iron intake and smoking status, and calculated the relative excess risk index (RERI) to assess the adherence of the data to an additive model of interaction and the multiplicative index (M) for the multiplicative interaction [27].

Given the heterogeneity in the methodology used in the studies included in the pooled analysis, we conducted a sensitivity analysis based on a two-stage approach. We first evaluated the association between total dietary iron intake and GC in each study, using the same multivariate logistic regression models of the pooled analysis. Next, we conducted a random-effects meta-analysis of these results [28]. To assess the contribution of individual studies to the overall results, we repeated the meta-analysis excluding one study at a time.

All of the statistical analyses were performed on STATA, version 16.1 (Stata Corp., College Station, TX, USA) [29]. A *p*-value lower than 0.05 was considered significant.

## 3. Results

The analysis included 16,905 subjects, comprising 4658 GC cases and 12,247 controls. Table 1 shows their distribution by study, sex, age, and major covariables. Cases were more frequently of low socioeconomic status (40.3%) than controls (34.4%). Also, cases were more frequently smokers (20.5% vs. 19.4%) and had higher caloric intake (median 2137.8 kcal/day vs. 2030.8 kcal/day) than controls. Overall, 21.2% of cases vs. 8.79% of controls reported a history of GC among first-degree relatives. Cases were more frequently in the top quartile of dietary iron (28.4%) and caloric intake (26.5%) than controls (23.7% and 21.1%, respectively).

The results of the primary multivariate analysis are summarized in Table 2. After adjustment for potential confounders, the apparent positive association between iron intake and risk of GC detected in the univariate comparison was reversed, and higher quartiles of dietary iron intake were associated with a significant reduced risk of GC (OR for Q2 = 0.88, 95% CI 0.78–0.99; for Q3 = 0.82, 95% CI 0.72–0.95; and for Q4 = 0.66, 95% CI 0.56–0.78) compared to the lowest quartile (Q1), with an OR of 0.88 (95% CI 0.83–0.93) for the increase in one quartile of iron intake. When considering total iron intake in study-specific quartiles, results were comparable (OR for 1 quartile increase = 0.90, 95% CI 0.85–0.95).

This pattern was confirmed in models that were further adjusted by quartiles of meat intake (i.e., OR = 0.90, 95% CI = 0.83–0.98 adjusting by meat intake, and OR = 0.91, 0.84–0.99, not adjusting for meat intake, based on eight studies with available information). The magnitude and direction of the association was maintained when adjusting for BMI, alcohol consumption, family history of GC, and vegetable and fruit intake.

The results of the sensitivity analysis excluding studies with >10% of missing data for iron, socioeconomic status and salt, also yielded similar findings.

The robustness of these results was further corroborated by the analysis excluding one study at a time (Supplementary Table S2).

Table 3 shows the results by GC subsite and histological type. The inverse association between iron and GC was consistent irrespective of cancer subsite. In particular, the OR for the highest quartile of iron intake was significantly reduced for both cardia (OR for Q4 vs. Q1 = 0.63, 95% CI = 0.47–0.86) and non-cardia GC (OR = 0.64, 95% CI = 0.51–0.81), and the OR for the increase in one quartile of intake showed a 15% reduction for cardia, and a 13% reduction for non-cardia GC. Iron was inversely related as well to intestinal (OR for Q4 vs. Q1= 0.65, 95% CI = 0.47–0.92) and diffuse type (OR = 0.46, 95% CI = 0.31–0.68). ORs for increase in one quartile were 0.88 (95% CI = 0.79–0.98) for the intestinal and 0.79 (95% CI = 0.69–0.89) for the diffuse type.

Stratified analyses according to sex, caloric intake, and smoking status are provided in Supplementary Table S3. There was no apparent effect modification by sex. The inverse association between dietary intake and risk of GC appeared somewhat stronger in the higher quartiles (Q3, Q4) of caloric intake vs. lower (Q1, Q2). The inverse association was also apparently stronger in magnitude for current smokers (OR for Q4 vs. Q1, 0.41; 95% CI, 0.27–0.61) compared to former (OR for Q4 vs. Q1, 0.75; 95% CI, 0.58–0.97) and never smokers (OR for Q4 vs. Q1, 0.80; 95% CI, 0.62–1.04). Hp status did not modify the effect of iron intake and GC.

When stratifying by control type, the negative association between iron intake and GC risk was evident in studies with population-based controls (OR for one quartile increase = 0.86, 95% CI = 0.81–0.91), compared to studies with hospital-based controls (OR = 0.97, 95% CI = 0.86–1.10).

**Figure 1:**
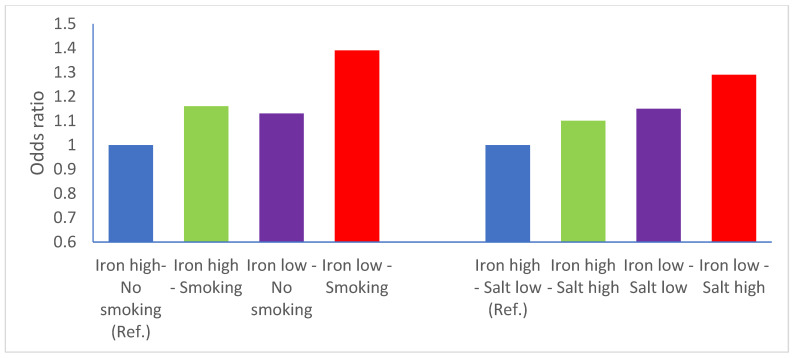
Interactions between iron and tobacco smoking and iron and salt intake—iron intake is categorized above (reference category) vs. below median intake. Odds ratios adjusted for study, sex, age, smoking status, socioeconomic status, calorie intake, salt intake. Ref = reference.

**Figure 2:**
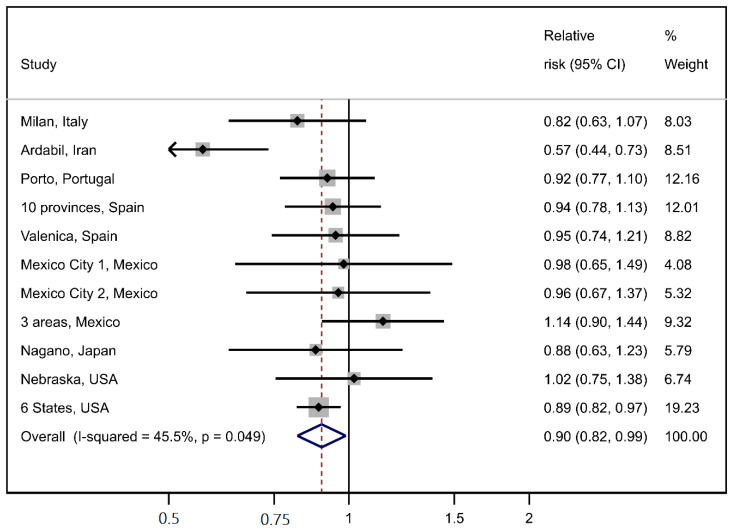
Meta-analysis of individual study results on the association between iron intake (one quartile increase) and gastric cancer.

The interaction between iron and smoking status was more compatible with a multiplicative model (M = 1.06, *p* = 0.49) rather than an additive model (RERI = 0.10, Figure 1, panel A). The interaction between iron and salt intake appeared to be compatible with both an additive model (RERI = 0.04) and a multiplicative one (M = 1.02, *p* = 0.75, Figure 1, panel B). The results of the meta-analysis of the study-specific OR for Q4 vs. Q1 of iron intake are reported in Figure 2. The meta-OR was 0.90 (95% CI 0.82–0.99), and the *p*-value of the test of heterogeneity was 0.049. These results are similar to those of the main analysis reported in Table 2. The robustness of these results was further corroborated by the analysis excluding one study at a time (Supplementary Table S2).

## 4. Discussion

We found an inverse association between total dietary iron and GC, corresponding to a 12% reduced risk of GC for the increase in one quartile of iron intake. The magnitude and direction of the association were similar between anatomical sub-sites (cardia and non-cardia) and histological type (intestinal and diffuse). The results were confirmed in a meta-analytic approach and in several sensitivity analyses. Moreover, the interaction between iron intake and smoking status appeared to follow a multiplicative model, suggesting independent opposing effects on GC, while that between iron salt intake did not distinguish between an additive and a multiplicative model.

Iron is not currently recognized among the dietary factors associated with GC. The last World Cancer Research Fund Report [5] did not mention iron among the nutritional items possibly linked to GC, while the International Agency for Research on Cancer has not evaluated iron in its Monographs program [30]. A meta-analysis that reported a positive association between iron intake and several cancers was inconclusive for GC [9]. Given the heterogeneous results on this topic, we provided a synthesis of evidence (Table 4, [8,12,17,31,32,33,34,35,36]). Among the studies in Table 4, Jakszyn and coauthors analyzed the European Prospective Investigation into Cancer and Nutrition (EPIC) and found a significant positive association between heme iron intake and GC [31], while several case-control studies described an inverse relationship when considering total dietary iron.

Additional evidence on the association between iron and cancer is provided by the studies of subjects affected by hemochromatosis, caused by mutations in the HFE gene, connoted by the progressive accumulation of iron because of altered iron transport proteins [37]. A nested case-control study within the EPIC cohort investigated the incidence of GC in subjects with HFE mutations, finding a functional polymorphism resulting in about a 30% excess risk of GC, with a stronger effect for the non-cardia and intestinal type [38]. Conversely, other authors find no relation between hemochromatosis and GC [39]. On the other hand, iron deficiency anemia enhances cancer risk, including for GC [40,41,42,43].

A potential modifier of the effect of iron is *Hp*, which is the main risk factor for GC [44]. *Hp* causes gastric atrophy and hence reduces acid secretion [44]. This may alter micronutrients bioavailability directly and by modifying gut microbiota [45]. Dietary heme iron has been reported to enhance the risk of GC in subjects infected with high-risk *Hp* strains, but not in those infected with low-risk ones [46].

A balanced diet provides a daily amount of 10 to 20 mg of iron, which corresponds to the intake of our study population (average intake 14.75 mg/day, 95% CI = 14.65–14.84) [32]. Among the two main types of iron, the heme fraction (organic) represents a small proportion of the total, which, however, can be more easily adsorbed than non-heme (inorganic) iron [32]. The latter represents the major fraction and is present in both animal and vegetable foods [32]. The proportion of heme iron contained in meat is minor compared to that of non-heme, so it seems that the direct link between red meat and GC is found irrespective of iron content. Indeed, this does not invalidate a negative association found with total iron.

Heme iron may contribute to carcinogenesis through increasing oxidative stress or by catalyzing the endogenous formation of N-nitroso compounds [32]. Given the lower bioavailability of non-heme iron, the total amount of iron intake should be adjusted for meat and vegetable intake when it is not possible to establish the food origin of the nutrient. To address this issue, we built models adjusted for either vegetable and fruit intake or meat intake: the association between iron and GC remained negative, suggesting the independent role played by iron. Similar results were also obtained when adjusting for alcohol drinking, which is a risk factor of GC [47].

This is one of the few studies investigating dietary iron by GC anatomical and histological categories, contributing to the definition of its effect on the specific GC types. Indeed, the negative association with iron intake was described for both cardia and non-cardia GC, with only a small difference between the two. Previous studies are limited, but they are consistent with ours, showing only minor differences in the role of dietary iron on cardia and non-cardia GC [31].

The dose-response relationship was confirmed for both diffuse and intestinal histological types. There are few comparable results given the difficulty in collecting histological data; however, dietary factors generally show similar results for intestinal and diffuse GC [21,48,49]. We identified only one study reporting the significant influence of dietary factors, including iron, on the intestinal but not the diffuse type [50]. These results can be due either to a real lack of difference in the subtypes’ vulnerability to the exposure, or to the lack of statistical power given the small number of histology specific results [49]. Our analysis was based on a large number of cases (1816 intestinal and 1151 diffuse), thus providing valuable insights. Additional studies would be needed to assess how nutritional factors are associated with the different subtypes of GC, which implies the effort required in collecting information on the GC type.

To our knowledge, this is the first study to investigate the interaction between iron and other risk factors of GC. Tobacco smoking and salt intake are reported to increase the risk of GC [51]. Our results suggest that iron acts independently from the other risk factors considered, with a protective effect towards GC also in smokers and in subjects with intermediate or high salt intake.

Iron intake is preferable to serum iron as a measure of the possible effect of the metal, as a loss of iron because of hemorrhage, malnutrition or malabsorption could reduce total serum levels [52]. This could particularly impair the use of serum iron to investigate this relationship in case-control studies where serum samples before cancer occurrence are unlikely to be available.

As we pooled data from different studies, dietary iron was not measured with the same instruments, which may result in exposure misclassification in pooled analysis. Moreover, some studies may have underestimated iron consumption because they might not have collected information on certain iron-containing foods. Additionally, the recalling of the frequency and quantity of specific foods consumed could have been influenced by the knowledge of disease status, although dietary assessment in many studies addressed the habit one or two years before diagnosis. The fact that the models were adjusted by study center accounts, at least in part, for this source of bias.

We partially addressed this potential limitation by adjusting the main model by meat, as well as by vegetables and fruit intake in separate analyses, which confirmed the negative association between iron and GC, suggesting an effect independent from vegetable or meat origin.

The pooled analysis was also mildly weakened by heterogeneity. This can be explained by the geographical and temporal variability among the pooled studies, and by differences in dietary exposure assessment. Additionally, *Hp* status was not considered in this analysis because of missing values in the majority of studies. Case–control studies have limited ability to measure *Hp* because blood samples of cases are obtained at GC diagnosis [12]. Indeed, *Hp* is subject to reverse causation, where the modification of cancer environment due to progressive mucosal damage leads to the clearance of the carcinogen agent; this could lead to differential misclassification of *Hp* status and underestimate the association; in particular it may be difficult to assess if negative cases had ever been positive (naïve) or not [53]. Besides this, up to 90% or more of non-cardia GC are due to Hp infection [44], reducing its role as potential confounder in our analysis, where non-cardia represents >70% of the total cases.

An additional limitation of our analysis was the lack of ability to distinguish the effect of heme and non-heme iron.

Our study consists of a large, pooled analysis including international studies, with detailed data available on different nutritional and lifestyle factors associated with GC. Iron intake was assessed by using calculations from FFQs, and the correspondent variables were built through accurate statistical analyses. Results were adjusted by important confounders for GC and corroborated by several sensitivity analyses. Information on subsite and histological types were available for many cases, leading to valuable results which have been rarely reported elsewhere. Moreover, we explored the interaction between iron and other factors, suggesting an independent role of dietary iron from tobacco smoking in the development of GC, with no prevailing model of interaction with salt intake. This is a new result, which contributes to providing a better knowledge on the possible role of iron on gastric carcinogenesis. Heterogeneity among the included studies was of borderline statistical significance. Thus, the fluctuations found among the study-specific results may be due to chance. Indeed, our analysis considered studies from different countries with potentially different dietary patterns, with no standardized criteria for collecting the exposures of interest. The results are strengthened by the fact that, as shown in the meta-analysis, the largest contribution to the overall association comes from a nested case-control study [26]: cohort studies are more likely to provide valid results than case-controls [12,54]. The fact that the apparent effect of iron intake was stronger in studies with population-based controls than in hospital-based ones provides additional support to the validity of our results.

In conclusion, we provided evidence of a possible inverse relationship between iron intake and GC. The association persisted when adjusting for vegetable and fruit intake, meat intake and other potential confounders, and appeared to be independent from smoking. The association was confirmed for both GC subsites and histological types. While the results should be interpreted with caution, given the difficulty in assessment of food intake, they provide evidence against a direct effect of iron in gastric carcinogenesis. Additional studies are needed to characterize this association; in particular, studies should include separate analyses of heme and non-heme iron in order to clarify any potential difference in their effect on GC risk. The mechanisms through which dietary iron might exert its effect on stomach carcinogenesis warrants further investigation, particularly since dietary intake represents a modifiable risk factor and holds promise for GC interception.

## Figures and Tables

**Table 1:** Distribution of cases of GC and controls according to study center, sex, age, and selected covariates *.

	Cases N (%)	Controls N (%)
**Total**	4658 (100.0)	12,247 (100.0)
Sex		
Male	3138 (67.4)	7343 (60.0)
Female	1520 (32.6)	4904 (40.0)
Age (years)		
≤55	871 (18.7)	2776 (22.7)
56–65	1048 (22.5)	3025 (24.7)
66–75	1819 (39.1)	4392 (35.9)
≥76	920 (19.8)	2050 (16.7)
Cigarette smoking		
Never	1876 (41.6)	5444(45,6)
Former	1710 (37.9)	4187 (35.1)
Current	927 (20.5)	2311 (19.4)
Alcohol drinking		
Never	1205 (28.7)	3144 (28.4)
Low	1464 (34.8)	4412 (39.8)
Intermediate	958 (22.8)	2533 (22.9)
High	576 (13.7)	985 (8.9)
Socio-economic status		
Low	1823 (40.3)	4142 (34.4)
Intermediate	1726 (38.1)	4555 (37.8)
High	976 (21.6)	3342 (27.8)
Salt consumption		
Low	2046 (45.4)	5080 (45.1)
Intermediate	1168 (25.9)	3149 (27.9)
High	1289 (28.6)	3046 (27.0)
Meat intake		
Q1	986 (24.2)	2357 (25.4)
Q2	900 (22.1)	2439 (26.3)
Q3	988 (24.2)	2359 (25.4)
Q4	1202 (29.5)	2131 (23.0)
Vegetables and fruit intake		
Low	1552 (36.5)	3406 (30.8)
Intermediate	1410 (33.2)	3773 (34.2)
High	1286 (30.3)	3869 (35.0)
Total caloric intake		
Q1	965 (21.8)	2853 (24.5)
Q2	1162 (26.3)	3303 (28.4)
Q3	1127 (25.5)	3030 (26.0)
Q4	1171 (26.5)	2456 (21.1)
Dietary iron intake		
Q1	969 (21.6)	2874 (24.5)
Q2	1083 (24.1)	3171 (27.0)
Q3	1160 (25.9)	2908 (24.8)
Q4	1274 (28.4)	2780 (23.7)
Family history of GC		
No	1170 (84.0)	5693 (93.0)
Yes	355 (16.0)	426 (7.0)
BMI		
18.5–24.9	1557 (40.6)	3608 (36.6)
25–29.9	1539 (40.1)	4297 (43.6)
30–34.9	585 (15.2)	1519 (15.4)
35–50	157 (4.1)	438 (4.4)
Anatomical site of GC		NA
Cardia	982 (30.3)	
Non-cardia	2258 (69.7)	
Histological type of GC		NA
Intestinal	1119 (59.6)	
Diffuse	791 (41.4)	

* Numbers may not add to the total because of missing values. Q, quartile; BMI, body mass index; GC, gastric cancer; NA, not applicable.

**Table 2:** Adjusted odds ratios and 95% confidence intervals of the association between dietary iron intake and other selected characteristics and gastric cancer.

Covariate	Adjusted OR	95% CI
**Dietary iron (quartiles)**		
Q1	Ref	
Q2	0.88	0.78–0.99
Q3	0.82	0.72–0.95
Q4	0.66	0.56–0.78
**Dietary iron (one quartile increase)**	0.88	0.83–0.93
**Tobacco smoking**		
Never	Ref	
Current	1.17	1.07–1.29
Former	1.22	1.09–1.36
**Socioeconomic status**		
Low	Ref	
Intermediate	0.65	0.59–0.72
High	0.52	0.46–0.58
**Calories (quartiles)**		
Q1	Ref	
Q2	1.20	1.06–1.36
Q3	1.28	1.11–1.49
Q4	1.57	1.33–1.87
**Salt intake**		
Low	Ref	
Medium	1.12	1.01–1.24
High	1.15	1.03–1.29
**Meat intake (quartiles)**		
Q1	Ref	
Q2	1.20	1.03–1.40
Q3	1.23	1.03–1.48
Q4	1.28	1.05–1.56
**BMI (kg/m^2^)**		
18.5–24.9	Ref	
25–29.9	0.77	0.70–0.84
30–34.9	0.84	0.74–0.95
35–50	0.85	0.69–1.05
**Alcohol drinking**		
Never	Ref	
Low	0.83	0.75–0.91
Intermediate	0.90	0.80–1.00
High	1.24	1.08–1.43
**Family history of GC**		
Negative	Ref	
Positive	2.37	2.01–2.79
**Vegetable and fruit intake**		
Low	Ref.	
Medium	0.80	0.73–0.88
High	0.69	0.61–0.76

OR, odds ratio, adjusted for study, sex, age, smoking status, socioeconomic status, caloric intake, salt intake. BMI, body mass index; CI, confidence interval; GC, gastric cancer; Q, quartile; Ref, reference category.

**Table 3:** Adjusted odds ratios and 95% confidence intervals of the association of total dietary iron intake by quartile, anatomical site and histological type of gastric cancer.

Dietary Iron Intake	Anatomical Site		Histological Type	
	Cardia OR (95%CI)	Non Cardia OR (95%CI)	Diffuse OR (95%CI)	Intestinal OR (95%CI)
**Quartiles**				
Q1	Ref	Ref	Ref	Ref
Q2	1.09 (0.86–1.39)	0.81 (0.69–0.96)	0.68 (0.51–0.91)	0.82 (0.64–1.04)
Q3	0.96 (0.73–1.25)	0.74 (0.61–0.90)	0.58 (0.42–0.81)	0.74 (0.56–0.98)
Q4	0.63 (0.47–0.86)	0.64 (0.51–0.81)	0.46 (0.31–0.68)	0.65 (0.47–0.92)
**One quartile increase**	0.85 (0.77–0.94)	0.87 (0.81–0.94)	0.79 (0.69–0.89)	0.88 (0.79–0.98)

OR, odds ratio, adjusted for study, sex, age, smoking status, socioeconomic status, caloric intake, salt intake. CI, confidence interval; Q, quartile; Ref, reference category.

**Table 4:** Selected other published studies on dietary iron and GC risk.

Reference	Iron Exposure	Comparison	OR	95% CI	Design
[31]	Dietary iron intake (heme)	Top vs. bottom quartile	1.67	1.20–2.34	Cohort
[32]	Dietary iron intake (heme)	Top vs. bottom quintile	0.83 cardia 0.72 non-cardia	0.53–1.30 0.48–1.08	Cohort
		100 μg increase	0.95 cardia 0.96 non-cardia	0.86–1.05 0.87–1.06	
[33]	Dietary iron intake	Top vs. bottom quartile	0.56	0.27–1.15	Case-control
[34]	Dietary iron intake (heme)	Top vs. bottom quintile (*p* for trend = 0.18)	4.83	NA	Cohort
[35]	Dietary iron intake	Top vs. bottom tertile (*p* for trend = 0.018)	0.65 total 0.81 heme 0.64 non heme	0.45–0.94 0.56–1.17 0.44–0.92	Case-control
[8]	Dietary iron intake (calorie adjusted)	Top vs. bottom quartile	1.05	0.60–1.85	Cohort
[12]	Dietary iron intake	Top vs. bottom quartile	1.71 total 1.99 heme	0.75–3.18 1.00–3.95	Case-control
[36]	Dietary ironintake	Top vs. bottom tertile (*p* for trend = 0.02)	0.41	0.19–0.89	Case-control

OR, odds ratio. CI, confidence interval.

## Data Availability

Individual data can be obtained from the StoP Project upon reasonable request and approval of the Principal Investigators of the original studies.

## Supplementary Materials

The following supporting information can be downloaded at: https://www.mdpi.com/article/10.3390/nu14122555/s1, Table S1: Selected characteristics of the studies included in the pooled analysis; Table S2: Odds ratios of GC for quartiles of dietary iron intake, excluding one study at a time; Table S3: Results of the analyses by sex, caloric intake, and tobacco smoking.

## References

[1] Sung H., Ferlay J., Siegel R.L., Laversanne M., Soerjomataram I., Jemal A., Bray F. (2021). Global Cancer Statistics 2020: GLOBOCAN Estimates of Incidence and Mortality Worldwide for 36 Cancers in 185 Countries. CA Cancer J. Clin..

[2] Helicobacter and Cancer Collaborative Group (2001). Gastric cancer and Helicobacter pylori: A combined analysis of 12 case control studies nested within prospective cohorts. Gut.

[3] Peleteiro B., Bastos A., Ferro A., Lunet N. (2014). Prevalence of Helicobacter pylori Infection Worldwide: A Systematic Review of Studies with National Coverage. Am. J. Dig. Dis..

[4] Ladeiras-Lopes R., Pereira A.K., Nogueira A., Pinheiro-Torres T., Pinto I., Santos-Pereira R., Lunet N. (2008). Smoking and gastric cancer: Systematic review and meta-analysis of cohort studies. Cancer Causes Control.

[5] World Cancer Research Fund International (2018). Diet, Nutrition, Physical Activity and Stomach Cancer.

[6] Vahid F., Davoodi S.H. (2020). Nutritional Factors Involved in the Etiology of Gastric Cancer: A Systematic Review. Nutr. Cancer.

[7] Abbaspour N., Hurrell R., Kelishadi R. (2014). Review on iron and its importance for human health. J. Res. Med. Sci..

[8] Cook M.B., Kamangar F., Weinstein S.J., Albanes D., Virtamo J., Taylor P.R., Abnet C.C., Wood R.J., Petty G., Cross A.J. (2012). Iron in Relation to Gastric Cancer in the Alpha-Tocopherol, Beta-Carotene Cancer Prevention Study. Cancer Epidemiol. Biomark. Prev..

[9] Fonseca-Nunes A., Jakszyn P., Agudo A. (2014). Iron and cancer risk—A systematic review and meta-analysis of the epidemiological evidence’. Cancer Epidemiol. Biomark. Prev..

[10] Noto J.M., Gaddy J.A., Lee J.Y., Piazuelo M.B., Friedman D.B., Colvin D.C., Romero-Gallo J., Suarez G., Loh J., Slaughter J. (2012). Iron deficiency accelerates Helicobacter pylori–induced carcinogenesis in rodents and humans. J. Clin. Investig..

[11] González C.A., Sala N., Rokkas T. (2013). Gastric Cancer: Epidemiologic Aspects. Helicobacter.

[12] Ward M.H., Cross A.J., Abnet C., Sinha R., Markin R.S., Weisenburger D.D. (2012). Heme iron from meat and risk of adenocarcinoma of the esophagus and stomach. Eur. J. Cancer Prev..

[13] International Agency for Research on Cancer (2018). Red Meat and Processed Meat: IARC Monographs on the Evaluation of Carcinogenic Risks to Humans.

[14] Pelucchi C., Lunet N., Boccia S., Zhang Z.F., Praud D., Boffetta P., Levi F., Matsuo K., Ito H., Hu J. (2015). The stomach cancer pooling (StoP) project: Study design and presentation. Eur. J. Cancer Prev..

[15] EFSA Draft Scientific Opinion: Scientific Opinion on Dietary Reference Values for Iron. https://www.efsa.europa.eu/sites/default/files/consultation/150526.pdf.

[16] Lucenteforte E., Scita V., Bosetti C., Bertuccio P., Negri E., La Vecchia C. (2008). Food Groups and Alcoholic Beverages and the Risk of Stomach Cancer: A Case-Control Study in Italy. Nutr. Cancer.

[17] Pakseresht M., Forman D., Malekzadeh R., Yazdanbod A., West R.M., Greenwood D.C., Crabtree J.E., Cade J. (2011). Dietary habits and gastric cancer risk in north-west Iran. Cancer Causes Control.

[18] Lunet N., Valbuena C., Lacerda Vieira A., Lopes C., David L., Carneiro F., Barros H. (2007). Fruit and vegetable consumption and gastric cancer by location and histological type: Case-control and meta-analysis. Eur. J. Cancer Prev..

[19] Castaño-Vinyals G., Aragones N., Pérez-Gómez B., Martín V., Llorca J., Moreno E., Altzibar J.M., Ardanaz E., de Sanjosé S., Jimenez-Moleon J.J. (2015). Population-based multicase-control study in common tumors in Spain (MCC-Spain): Rationale and study design. Gac. Sanit..

[20] Santibañez M., Alguacil J., de la Hera M.G., Navarrete-Muñoz E.M., Llorca J., Aragonés N., Kauppinen T., Vioque J., for the PANESOES Study Group (2011). Occupational exposures and risk of stomach cancer by histological type. Occup. Environ. Med..

[21] Hernández-Ramírez R.U., Galván-Portillo M.V., Ward M.H., Agudo A., González C.A., Oñate-Ocaña L.F., Herrera-Goepfert R., Palma-Coca O., López-Carrillo L. (2009). Dietary intake of polyphenols, nitrate and nitrite and gastric cancer risk in Mexico City. Int. J. Cancer.

[22] Ward M.H., Bravo-Alvarado J., López-Carrillo L., López-Cervantes M., Ramírez-Espitia A. (1999). Nutrient intake and gastric cancer in Mexico. Int. J. Cancer.

[23] López-Carrillo L., López-Cervantes M., Robles-Díaz G., Ramírez-Espitia A., Mohar-Betancourt A., Meneses-García A., López-Vidal Y., Blair A. (2003). Capsaicin consumption, Helicobacter pylori positivity and gastric cancer in Mexico. Int. J. Cancer.

[24] Machida-Montani A., Sasazuki S., Inoue M., Natsukawa S., Shaura K., Koizumi Y., Kasuga Y., Hanaoka T., Tsugane S. (2004). Association of Helicobacter pylori infection and environmental factors in non-cardia gastric cancer in Japan. Gastric Cancer.

[25] Ward M.H., Sinha R., Heineman E.F., Rothman N., Markin R., Weisenburger E.E., Correa P., Zahm S.H. (1997). Risk of adenocarcinoma of the stomach and esophagus with meat cooking method and doneness preference. Int. J. Cancer.

[26] Schatzkin A., Subar A.F., Thompson F.E., Harlan L.C., Tangrea J., Hollenbeck A.R., Hurwitz P.E., Coyle L., Schussler N., Michaud D.S. (2001). Design and serendipity in establishing a large cohort with wide dietary intake distributions: The National Institutes of Health-American Association of Retired Persons Diet and Health Study. Am. J. Epidemiol..

[27] Vander Weele T.J., Knol M.J. (2014). A Tutorial on Interaction. Epidemiol. Methods.

[28] Der Simonian R., Laird N. (1986). Meta-analysis in clinical trials. Control Clin. Trials.

[29] StataCorp (2019). Stata Statistical Software: Release 16.

[30] Report of the Advisory Group to Recommend Priorities for IARC Monographs during 2015–2019. https://monographs.iarc.who.int/wp-content/uploads/2018/08/14-002.pdf.

[31] Jakszyn P., Bingham S., Pera G., Agudo A., Luben R., Welch A., Boeing H., del Giudice G., Palli D., Saieva C. (2006). Endogenous versus exogenous exposure to N -nitroso compounds and gastric cancer risk in the European Prospective Investigation into Cancer and Nutrition (EPIC-EURGAST) study. Carcinogenesis.

[32] Cross A.J., Freedman N.D., Ren J., Ward M.H., Hollenbeck A.R., Schatzkin A., Sinha R., Abnet C. (2011). Meat Consumption and Risk of Esophageal and Gastric Cancer in a Large Prospective Study. Am. J. Gastroenterol..

[33] Pelucchi C., Tramacere I., Bertuccio P., Tavani A., Negri E., La Vecchia C. (2008). Dietary intake of selected micronutrients and gastric cancer risk: An Italian case-control study. Ann. Oncol..

[34] Lee D.H., Anderson K.E., Folsom A.R., Jacobs D.R. (2005). Heme iron, zinc and upper digestive tract cancer: The Iowa Women’s Health Study. Int. J. Cancer..

[35] Tran T., Gunathilake M., Lee J., Choi I., Kim Y.-I., Kim J. (2021). The Associations of Dietary Iron Intake and the Transferrin Receptor (*TFRC*) rs9846149 Polymorphism with the Risk of Gastric Cancer: A Case–Control Study Conducted in Korea. Nutrients.

[36] Cornée J., Pobel D., Riboli E., Guyader M., Hémon B. (1995). A case-control study of gastric cancer and nutritional factors in Marseille, France. Eur. J. Epidemiol..

[37] Crownover B.K., Covey C.J. (2013). Hereditary hemochromatosis. Am. Fam. Physician.

[38] Mikhailova S.V., Babenko V., Ivanoshchuk D.E., Gubina M.A., Maksimov V.N., Solovjova I.G., Voevoda M.I. (2016). Haplotype analysis of the HFE gene among populations of Northern Eurasia, in patients with metabolic disorders or stomach cancer, and in long-lived people. BMC Genet..

[39] Lagergren K., Wahlin K., Mattsson F., Alderson D., Lagergren J. (2016). Haemochromatosis and gastrointestinal cancer. Int. J. Cancer.

[40] Prá D., Rech Franke S.I., Pegas Henriques J.A., Fenech M. (2009). A possible link between iron deficiency and gastrointestinal carcino-genesis. Nutr. Cancer.

[41] Broitman S.A., Velez H., Vitale J.J. (1981). A Possible Role of Iron Deficiency in Gastric Cancer in Colombia. Adv. Exp. Med. Biol..

[42] Hung N., Shen C.-C., Hu Y.-W., Hu L.-Y., Yeh C.-M., Teng C.-J., Kuan A.-S., Chen S.-C., Chen T.-J., Liu C.-J. (2015). Risk of Cancer in Patients with Iron Deficiency Anemia: A Nationwide Population-Based Study. PLoS ONE.

[43] Hudak L., Jaraisy A., Haj S., Muhsen K. (2017). An updated systematic review and meta-analysis on the association between Helico-bacter pylori infection and iron deficiency anemia. Helicobacter.

[44] International Agency for Research on Cancer (1994). Infection with Helicobacter pylori: IARC Monographs on the Evaluation of Carcinogenic Risks to Humans, Vol. 61, Schistosomes, Liver Flukes and Helicobacter pylori.

[45] Bielik V., Kolisek M. (2021). Bioaccessibility and Bioavailability of Minerals in Relation to a Healthy Gut Microbiome. Int. J. Mol. Sci..

[46] Epplein M., Zheng W., Li H., Peek R.M., Correa P., Gao J., Michel A., Pawlita M., Cai Q., Xiang Y.-B. (2014). Diet, Helicobacter pylori strain-specific infection, and gastric cancer risk among Chinese men. Nutr. Cancer..

[47] Ferro A., Morais S., Rota M., Pelluchi C., Bertuccio P., Bonzi R., Galeone C., Zhang F.-Z., Matsuo K., Ito H. (2018). Alcohol intake and gastric cancer: Meta-analyses of published data versus individual par-ticipant data pooled analyses (StoP Project). Cancer Epidemiol..

[48] Buiatti E., Palli D., Bianchi S., Decarli A., Amadori D., Avellini C., Cipriani F., Cocco P., Giacosa A., Lorenzini L. (1991). A case-control study of gastric cancer and diet in Italy. III. Risk patterns by histologic type. Int. J. Cancer.

[49] Buckland G., Agudo A., Luján L., Jakszyn P., Bueno-de-Mesquita H.B., Palli D., Boeing H., Carneiro F., Krogh V., Sacerdote C. (2010). Adherence to a Mediterranean diet and risk of gastric adenocarcinoma within the Eu-ropean Prospective Investigation into Cancer and Nutrition (EPIC) cohort study. Am. J. Clin. Nutr..

[50] Harrison L.E., Zhang Z.F., Karpeh M.S., Sun M., Kurtz R.C. (1997). The role of dietary factors in the intestinal and diffuse histologic subtypes of gastric adenocarcinoma: A case-control study in the U.S. Cancer.

[51] Machlowska J., Baj J., Sitarz M., Maciejewski R., Sitarz R. (2020). Gastric Cancer: Epidemiology, Risk Factors, Classification, Genomic Characteristics and Treatment Strategies. Int. J. Mol. Sci..

[52] Rosania R., Chiapponi C., Malfertheiner P., Venerito M. (2016). Nutrition in Patients with Gastric Cancer: An Update. Gastrointest. Tumors.

[53] Collatuzzo G., Pelucchi C., Negri E., López-Carrillo L., Tsugane S., Hidaka A., Hamada G.S., Hernández-Ramírez R.U., López-Cervantes M., Malekzadeh R. (2021). Exploring the interactions between Helicobacter pylori (Hp) infection and other risk factors of gastric cancer: A pooled analysis in the Stomach cancer Pooling (StoP) Project. Int. J. Cancer.

[54] Rothman K.J., Vander Weele T.J., Lash T.L., Lash T.L., Vander Weele T.J., Haneuse S., Rothman K.J. (2021). Cohort Studies. Modern Epidemiology.

