# Supplementary material for: Inverse Association between Dietary Iron Intake and Gastric Cancer: A Pooled Analysis of Case-Control Studies of the Stop Consortium
Source: Nutrients. 2022 Jun 20;14(12):2555. doi: 10.3390/nu14122555 (PMC9228527; doi:10.3390/nu14122555)
Supplement: Supplementary file 1 [file nutrients-14-02555-s001.zip › Supplementary tables.pdf]

Supplementary Table S1. Selected characteristics of the studies included in the pooled analysis.

Reference	Area, country	Study period	Cases (n)	Controls (n)	Study design
Lucenteforte et al., 2008 [16]	Milan, Italy	1997-2007	230	547	Hospital-based
Pakseresht et al., 2011 [17]	Ardabil, Iran	2005-2007	286	304	Population-based
Lunet et al., 2007 [18]	Porto, Portugal	1999-2006	692	1667	Population-based
Castano-Vinyals et al., 2015 [19]	10 provinces, Spain	2008-2012	441	3440	Population-based
Santibanez et al., 2012 [20]	Valencia, Spain	1995-1999	401	455	Hospital-based
Hernandez -Ramirez et al., 2009 [21]	Mexico City, 1 Mexico	2004-2005	248	478	Population-based
Lopez-Carrillo et al., 1999 [22]	Mexico City 2, Mexico	1989-1990	220	752	Population-based
Lopez-Carrillo et al., 2003 [23]	3 areas, Mexico	1994-1996	234	468	Hospital-based
Machida-Montani et al., 2004 [24]	Nagano, Japan	1998-2002	153	303	Hospital-based
Ward et al., 1997 [25]	Nebraska, USA	1988-1993	170	502	Population-based
Schatzkin et al., 2001 [26]	6 States, USA	1995-1996	1583	3331	Nested in a cohort

Supplementary Table S2. Odds ratio of GC for quartiles of dietary iron intake, excluding one study at a time.

Study excluded	Dietary iron intake (quartile)	OR	95% CI
Milan, Italy	Q1	Ref	
	Q2	0.87	(0.76-0.98)
	Q3	0.82	(0.71-0.95)
	Q4	0.66	(0.56-0.78)
Ardabil, Iran	Q1	Ref	
	Q2	0.90	(0.80-1.02)
	Q3	0.88	(0.76-1.02)
	Q4	0.73	(0.61-0.87)
Porto, Portugal	Q1	Ref	
	Q2	0.87	(0.77-0.99)
	Q3	0.83	(0.71-0.96)
	Q4	0.66	(0.56-0.79)
10 provinces, Spain	Q1	Ref	
	Q2	0.85	(0.75-0.97)
	Q3	0.78	(0.67-0.91)
	Q4	0.65	(0.55-0.77)
Valencia, Spain	Q1	Ref	
	Q2	0.91	(0.80-1.03)
	Q3	0.81	(0.70-0.94)
	Q4	0.65	(0.55-0.77)
Mexico City, 1 Mexico	Q1	Ref	
	Q2	0.91	(0.81-1.04)
	Q3	0.89	(0.77-1.03)
	Q4	0.68	(0.56-0.79)
Mexico City 2, Mexico	Q1	Ref	
	Q2	0.88	(0.77-0.99)
	Q3	0.82	(0.71-0.94)
	Q4	0.67	(0.57-0.80)
3 areas, Mexico	Q1	Ref	
	Q2	0.86	(0.76-0.98)
	Q3	0.81	(0.70-0.94)
	Q4	0.64	(0.54-0.76)
Nagano, Japan	Q1	Ref	
	Q2	0.88	(0.77-1.00)
	Q3	0.82	(0.71-0.95)
	Q4	0.66	(0.56-0.79)
Nebraska, USA	Q1	Ref	
	Q2	0.88	(0.77-0.99)
	Q3	0.81	(0.70-0.93)
	Q4	0.65	(0.55-0.77)
6 States, USA	Q1	Ref	
	Q2	0.83	(0.71-0.97)
	Q3	0.78	(0.65-0.94)

	Q4	0.63	(0.50-0.79)

OR, Odds ratio, adjusted for study, sex, age, smoking status, socioeconomic status, caloric intake, salt intake

CI, confidence interval; Q, quartile ; Ref, reference category

Supplementary Table S3: Results of the analyses stratified by sex, caloric intake and tobacco smoking

**Stratification by**	**Total dietary iron OR (95% CI)**				
	**Q1**	**Q2**	**Q3**	**Q4**	**One quartile increase**
**Sex***					
Male	Ref	0.83 (0.71-0.97)	0.76 (0.64-0.91)	0.60 (0.49-0.74)	0.85 (0.80-0.91)
Female	Ref	0.93 (0.77-1.13)	0.89 (0.70-1.13)	0.77 (0.57-1.04)	0.92 (0.84-1.02)
**Calories intake (quartiles)****					
Q1	Ref	1.06 (0.87-1.29)	0.91 (0.68-1.22)	0.81 (0.46-1.42)	0.97 (0.86-1.08)
Q2	Ref	0.92 (0.74-1.14)	0.88 (0.69-1.13)	0.84 (0.61-1.16)	0.95 (0.86-1.04)
Q3	Ref	0.73 (0.51-1.04)	0.77 (0.54-1.12)	0.53 (0.36-0.79)	0.85 (0.76-0.94)
Q4	Ref	0.82 (0.42-1.60)	0.55 (0.29-1.03)	0.45 (0.24-0.84)	0.77 (0.68-0.88)
**Tobacco smoking*****					
Never	Ref	0.96 (0.80-1.16)	0.91 (0.73-1.13)	0.80 (0.62-1.04)	0.93 (0.86-1.01)
Former	Ref	0.88 (0.71-1.08)	0.88 (0.70-1.10)	0.75 (0.58-0.97)	0.92 (0.84-1.00)
Current	Ref	0.77 (0.58-1.03)	0.64 (0.46-0.90)	0.41 (0.27-0.61)	0.75 (0.66-0.85)

* OR, Odds ratios, adjusted for study, age, smoking status, socioeconomic status, calorie intake, salt intake
** OR, Odds ratios, adjusted for study, sex, age, smoking status, socioeconomic status, salt intake
*** OR, Odds ratios, adjusted for study, sex, age, socioeconomic status, calorie intake, salt intake
CI, confidence interval; Q, quartile; Ref, reference category
